# Mindfulness beyond secularization: Beliefs across meditators and non-meditators reflect a consensus on personal development over health and spirituality

**DOI:** 10.1371/journal.pone.0331021

**Published:** 2025-09-08

**Authors:** Philippine Chachignon, Emmanuelle Le Barbenchon, Cécile Dantzer, Lionel Dany

**Affiliations:** 1 LPS, Aix Marseille Univ, Aix-en-Provence, France; 2 Université de Bordeaux, LabPsy U.R., Bordeaux, France; University of Zanjan, IRAN, ISLAMIC REPUBLIC OF

## Abstract

**Background:**

Mindfulness meditation (MM), originating from spiritual traditions but widely promoted as a secular and beneficial practice, is increasingly debated due to potential adverse effects, ethical concerns, and its ties with neoliberal imperatives, challenging its image as a universal remedy. Beliefs about MM strongly influence its reception, usage, and effects but remain understudied, especially in comparing meditators and non-meditators. Understanding these beliefs is key to clarifying how lay perceptions align or diverge from scientific frameworks and to grasp individuals’ expectations and motivations, notably in clinical contexts. Existing research often overlooks belief content or comparisons between meditators and non-meditators.

**Objective:**

This study explored the content of beliefs about MM, identified missing elements, and compared meditators’ and non-meditators’ beliefs. Associations with sociodemographic, motivational, health, and psychological variables were examined.

**Methods:**

167 participants (105 meditators) completed an online survey producing five words linked to MM, rated for valence, plus questionnaires on motivation, beliefs, and personal characteristics.

**Results:**

817 free associations were collected; 65% were positive. Hierarchical classification identified five belief categories: “Body-based Relaxation,” “Stereotyped Descriptions,” “Psychological and Affective Well-Being,” “Focus on Inner-Self,” and “Experience of MM.” Four categories were shared by both groups, with meditators showing more precise, experiential understanding. Beliefs varied with sociodemographic, health, and psychological factors. Core aspects in MM like attention, acceptance, health, collective dynamics and ethical concerns were largely absent. Notably, spirituality was not integrated into the beliefs about MM.

**Conclusions:**

Findings emphasize MM as a self-regulatory and personal development tool shaped by social, psychological and behavioural factors. Recognizing both current and potential MM users’ beliefs can improve tailoring of mindfulness interventions and encourage instructors to address ethical and adverse aspects openly, fostering more informed, responsible practice.

## Introduction

Mindfulness has long heightened the imaginaries of Westerners and has now integrated the daily routines of a significant portion of the population (e.g., around 7% of the US population practice Mindfulness Meditation (MM) [1,2]). From its original translation from the Pali word “Sati” by English Buddhologist Thomas Williams Rhys Davids in the late 19th century [3], to smart-phone apps such as Headspace or Calm, it has permeated different lay and scientific areas, such as spirituality, counterculture, health and personal development. In the 1970s, concurrently with the efforts of Socially Engaged Buddhist Thich Nhat Hanh to popularize MM in the West and anchor its spiritual dimensions, through monasteries, books and talks emphasizing ethics, compassion and non-violence [3], physician Jon Kabat-Zinn adapted MM into a secular therapeutic protocol known as Mindfulness-Based Stress Reduction (MBSR). Utilizing MM to address psychological and somatic difficulties, he contributed, along with other Western pioneers in the field [4], to forging its status as a health and scientific object fitted for institutionalization [5].

Due to mindfulness’s diverse epistemologies, as we briefly outlined, the scientific community continues to grapple with the challenge of delineating such multi-faceted concepts as mindfulness and MM [6]. Notably, MM is not exclusive to secular or Buddhist contexts but also appears in other religious traditions, including Sufism and Christian prayer. In the Islamic tradition, various meditative practices expand upon elements of ritual prayer. Sufi Muslims, for example, explore these practices independently to foster introspection, dissolve the ego’s illusions, and purify the heart [7]. The dominant secular definition of MM, consisting in “paying attention in a particular way, on purpose, in the present moment, and non-judgmentally” [8] (p. 4), has been serving as a conceptual basis for the majority of the tens of thousands of scientific publications [9]. Mostly documenting intra-individual effects, studies indicate that Mindfulness-based interventions (MBIs) can effectively reduce anxiety and mood problems, and improve mental health (see [10,11] for meta-analyses). Yet, recent meta-analyses showing minimal or adverse effects (e.g., [12,13]) have come to challenge the idea that MM is universally beneficial and safe. These adverse effects may include the exacerbation of traumatic stress symptoms—such as dissociation, intrusive memories, and emotional arousal—particularly among vulnerable individuals [14]. A range of adverse experiences has also been documented, including somatic (e.g., gastrointestinal issues), psychiatric (e.g., anxiety, depression, suicidal ideation), and cognitive symptoms (e.g., anomalies in thinking), with anxiety (33%), depression (27%), and cognitive disturbances (25%) being among the most frequently reported [12]. These findings call for a better understanding of how MM is approached and perceived by the public, especially as its use expands beyond clinical supervision. Meanwhile, the popularity of MM, including among the general population, continues to grow [15] which is noteworthy from a socio-cognitive perspective. Added to the plethora of MBIs in organizational and healthcare settings, the current availability of self-help MM smartphone apps and the presence of images of Buddhism and meditators in the media (e.g., in advertisement and films [16]) one may wonder to what extent these socio-cultural and symbolic universes have contributed to shaping the knowledge, beliefs and expectancies in the general population.

While the semblance of a honeymoon period may be palpable in the public sphere, within academic domains, emerging areas of tension are becoming apparent. Indeed, the current hype and ubiquity of MM has piqued the curiosity of numerous critical scholars, who have drawn attention to its ideological entanglements with neoliberal capitalism. This body of work has emphasized how MM contributes to individual responsibilization, the psychologization of systemic issues [17–20], and its transformation into both a commodity and a technology of the self [21–23]. Others have argued that the ethical foundations of mindfulness were lost during its secularization, with MBIs primarily focused on symptom relief rather than incorporating ethical principles such as right speech, right conduct, or right livelihood [24–26]. In line with these critiques, [27]—among the first contributors to this broader movement—argue that mindfulness, originally grounded in Eastern traditions promoting detachment from self-preoccupations and rooted in universal compassion, may clash with Western ideals of productivity and consumerism. As a result, it risks being commodified as a tool for personal efficiency, self-improvement and stress reduction, at the expense of collective transformation, particularly within organizational theory (for a review, see [28]). These tensions highlight the need to investigate how the practice is symbolically constructed in everyday discourse, beyond its academic and clinical framings. These concerns have gradually led to the emergence of Second-Generation MBIs, which reintroduce spiritual and ethical dimensions into mindfulness-based programs [29] though such approaches remain relatively uncommon. To our knowledge, only a few empirical studies have investigated the ideological implications of mindfulness. While some have linked trait mindfulness to reduced materialism, lower system justification, and greater support for progressive values (e.g., [30–32]), others suggest that mindfulness practice may relate differently. For instance, [33] found it was indirectly associated with higher economic system justification, in contrast with trait mindfulness. Since all these insights originate from the academic sphere, exploring whether the criticisms have penetrated common-sense knowledge would provide a pivotal perspective in understanding the object. It seems also crucial for both researchers and clinicians, as these beliefs shape how individuals engage with MM and what they expect from it.

Accordingly, it is timely to explore the meaning of, and the beliefs about MM, in the general population. Particularly, it is pertinent to compare these beliefs between individuals who practice MM and those who do not. In this respect, to our knowledge, the (limited) empirical studies on the beliefs associated with MM fail to explore the relationship between MM practice (or not) and common-sense knowledge. On one hand, a stream of research examines the beliefs about MM in the general population, where MM is considered socially acceptable, positively rated, and thought to be related to stress reduction and engagement in exploring lived experiences [1,34–37]. On the other hand, some scholars delve into the social psychological variables determining the use of MM. For instance, meditators differ from non-meditators in terms of health status, with higher levels of physical activity and alcohol consumption, exhibiting more depression and chronic pain and tending to use complementary therapies more frequently [1]. Motivations to practice include self-regulation, pain management, improved interpersonal relationships and spirituality [38]. Investigations into attitudes towards complementary and alternative medicine (such as MM) have shown that beliefs can be forged by social psychological factors such as health status or gender, as well as expectancies and motivations (e.g., [39]). Nonetheless, studies on beliefs associated with MM either overlook comparing populations of meditators and non-meditators or exploring the content(s) of the beliefs.

Understanding how mindfulness is perceived outside academic and clinical circles—particularly among those who engage with it versus those who do not—thus appears both timely and necessary. It allows us to grasp how the object circulates in the social world, how it is symbolically invested, and to what extent its current cultural penetration aligns with or diverges from its original intentions and ethical roots. Despite the growing body of research on mindfulness meditation (MM), few empirical studies have specifically examined beliefs about MM (e.g., [36,40,41]). To our knowledge, none has investigated how these beliefs differ between those who practice MM and those who do not—particularly regarding motivations, expectancies, and social or health-related factors. Moreover, existing research often overlooks the qualitative content of these beliefs and the potential absence of certain aspects within common-sense knowledge [42]. This gap limits our understanding of how mindfulness is socially constructed and represented across different population groups. Accordingly, this study aims to fill this gap by combining an analysis of the spontaneous beliefs about MM expressed by meditators and non-meditators, alongside an examination of how socio-demographic, health, psychological, and behavioural variables shape these beliefs. This also allows us to examine whether certain social positions are more conducive to specific understandings of mindfulness. Indeed, mindfulness practice tends to be more prevalent among a relatively homogeneous population—namely, White, educated women from middle- to upper-class backgrounds [1]. Such a sociological profile creates an archetype of the typical meditator, and raises the question of whether different social locations may give rise to different beliefs about MM.

### Aims

Specifically, our objectives are twofold. First, to explore the content of beliefs about MM in both groups through free association techniques, paying attention not only to what is present but also to what is notably absent from these beliefs. Second, to investigate how various factors, including MM practice status, influence the nature and structure of these beliefs. By doing so, this research sheds light on how psychological practices circulate socially and become invested with meaning—offering valuable insights for both clinical communication and the psychology of mindfulness. Accordingly, we formulate three hypotheses. First, we expect that the beliefs about MM will predominantly focus on positive aspects such as health, psychological well-being, and spirituality, reflecting previous findings from lay evaluations of MM. Second, we hypothesize that individuals who practice MM will express beliefs that are more detailed and accurate regarding the processes and goals of MM, owing to their experiential familiarity. Third, in an exploratory manner, we examine whether differences in health status, motivations, attitudes towards health behaviour, and socio-demographic factors predict variations in MM beliefs, assuming that individuals’ positioning in the social space influences how they conceptualize such practices.

## Methods

### Participants and procedure

This correlational study was conducted using an on online survey administered via LimeSurvey, where participants provided written informed consent in the first page by checking a box. The study protocol was approved by the Ethics Committee of Aix-Marseille University (n°24-02-15-02). The data was collected from February 15, 2024, to February 25, 2024. The research was conducted in a French sample (*N* = 185) of meditators and non-meditators recruited via social networks and MM networks. Eighteen participants did not properly complete the beliefs items and were therefore deleted from the total sample due to missing data. Additionally, they did not provide information on gender and age variables. These participants were not statistically different in terms of meditation practice than the rest of the sample (*χ²*(1) = 3.09, *p* = .07). Therefore, the total sample was 167 with 105 participants practicing MM (62.87% of the sample). Participants were considered as meditators if they reported having practiced mindfulness meditation and provided details regarding recent practice (e.g., length, frequency, seniority of the practice, type of practices and types of resources to practice). They practiced an average of 23.9 minutes per session, with an average seniority of 34.8 months. The meditators subsample included 83 females (*M*_age_ = 33.7 years, *SD =* 14.4 years) and 67% of participants reported having an undergraduate degree or higher). The non-meditators group (n = 62) included 47 females, (*M*_age_ = 29.7 years, *SD =* 11.8 years) and 69% reported having an undergraduate degree or higher). Using the global sample median age of 29 (*M*_age_ = 32.3, *SD* = 13.6), we dichotomized the variable into two groups: participants younger than 29 (age_inferior) and those aged 29 and above (age_superior). Participants who were exactly 29 years old were classified in the age_superior group. Meditators and non-meditators did not differ on socio-demographic criteria except for occupational category *χ²*(5) = 11.9, *p* = .037, and *χ²*_*cor*_(5) = 11.9, *p* = .037. A sensitivity test, conducted on G*power for *T*-Tests with independent means, a power set at.80 and an *α*-level of.05, indicated that the smallest effect that could be detected is of.40.

### Materials

The survey consisted of two parts: free associations that allow direct and easy access to common-sense knowledge [43], and a questionnaire. These were incorporated into a single survey designed in a way that the free associations material appeared first.

Beliefs about MM were collected *via* free associations in response to the questions “What are the first 5 words that come to your mind when you think about Mindfulness?”. Participants indicated the valence of each of the given words: positive, neutral or negative. To identify the characteristics of the participants, we collected 4 families of variables that were associated within the qualitative analysis.

1) **Socio-demographic characteristics** (age, gender, and socio-economic status such as occupational category and level of education).
2) **Meditation practice information** (practice, seniority of the practice and actual or imagined self-motivation to practice).
3) **Health and health behaviours**: a) Chronic illness; b) Tobacco use, alcohol drinking, and cannabis use: Since these three behaviours were significantly correlated, we calculated a global index referred to as the Tobacco-Alcohol-Cannabis (TAC) score. If the participant reported at least two out of these three behaviours with a score of 2 or higher (on a scale ranging from 0 = never to 4 = regularly), their overall behaviour was categorized as “unfavourable” to health. Otherwise, it was considered “favourable”; c) Food consumption including vegetables or fruits, fast-food, sweets or pastries, and sugary drinks. These last three variables were pooled in a global “Nutrition” score; non-alcoholic beverage consumption such as tea, coffee and energy drinks; d) sleeping hours; e) physical activity. All these items are derived from questions used in general population surveys such as those conducted by Santé Publique France [44].
4) **Beliefs and attitudes towards the practice** (see “The beliefs about MM” section below). Most responses for these variables were dichotomized: they were categorized as low or high scores based on the median in order to integrate and analyse them alongside the free associations; a) **Self-Motivations for MM practice.** The motivations for practice were measured among both meditators and non-meditators. Non-meditators were asked to imagine for what reasons they would practice. This was done using a unidimensional *ad hoc* scale comprising 12 items on a 5-point Likert scale ranging from 1 (strongly disagree) to 5 (strongly agree). Example items were “To have a better self-esteem”, or “To get closer to or reach the ideal version of myself”. Internal consistency of this measure was good, Cronbach’s *α* = .80; b) **The Beliefs about MM.** The beliefs about MM items were adapted from two questionnaires to suit the specific context of the study: The Credibility/expectancy questionnaire [45] and a questionnaire about complementary medicines [46]. A total of 26 items were asked for agreement on a 7-points scale.

Since beliefs and attitudes about health can be theoretically distinct yet empirically correlated, we used an exploratory factor analysis (EFA) with oblique rotation, which allows for intercorrelations among these dimensions [47], in order to investigate the latent structure of beliefs about MM. Prior to conducting the EFA on the 26 items, sampling adequacy was assessed. The overall Kaiser-Meyer-Olkin (KMO) measure of sampling adequacy was 0.87, indicating “meritorious” sampling adequacy [48]. Bartlett’s test of sphericity was significant, *χ²*(df = 351) = 2199.75, *p* < .001, indicating that the data were factorable [49].

Oblimin rotation was performed to determine a simple and interpretable factor structure and since we expected correlations between factors. The number of factors to retain was determined using a combination of eigenvalues (> 1 criterion), scree plot analysis, and parallel analysis [50]. These criteria suggested the retention of 3 factors. Nine items were removed for low communality (<.30) and one because it was redundant with another item.

A second EFA was performed on the 16 final items using the maximum likelihood extraction method with oblimin rotation, allowing for correlated factors. Items with loadings of at least.30 were considered salient [51], and cross-loadings were examined carefully. Items with cross-loadings < .20 apart or loadings below threshold were considered for removal or re-evaluation. Bartlett’s test of sphericity was still significant, *χ²*(df = 120) = 1515.77, *p* < .001, indicating that the data were factorable.

The final three-factor solution (see Table 1 for the results of the EFA) accounted for 47.6% of the total variance. The factor loadings are presented in Table 1. Inter-factor correlations ranged from −0.53 to 0.52, suggesting moderate conceptual overlap among the factors. Communalities were generally acceptable (above.30), indicating that the items were well represented by the factor structure. Internal consistency for each factor was assessed using Cronbach’s alpha. Reliability coefficients ranged from *α* = .84 to.89, indicating good reliability for each scale [52].

**Table 1:** Loadings of beliefs items (exploratory factorial analysis, Oblimin rotation).

	Harmful and inappropriate 19.3% *α* = 0.88	Perceived efficacy 19.1% *α* = 0.89	Placebo effect 18% *α* = 0.84	Communality
C1. The practice of mindfulness meditation can be adapted to the difficulties people encounter.	−0.344	0.450		**0.43**
C2. Mindfulness meditation can effectively reduce health problems.		0.937		**0.82**
C3. Mindfulness meditation effectively reduces symptoms that people may face.		0.618		**0.52**
C4. I would recommend mindfulness meditation to a friend		0.349	−0.405	**0.54**
C5. Mindfulness meditation is effective in reducing health problems.		0.906		**0.82**
R2. Mindfulness meditation is somewhat unscientific.			0.735	**0.49**
R4. Mindfulness meditation is harmful to people’s health	0.894			**0.69**
R5. People practicing mindfulness meditation almost never get better.	0.653			**0.63**
R6. The effects of mindfulness meditation are just a placebo effect.	0.323		0.526	**0.54**
R7. Mindfulness meditation is not rewarding because the treatment takes too long.	0.319		0.433	**0.43**
R12. Healthcare professionals should adopt mindfulness meditation to complement the effectiveness of conventional medical treatments.	−0.394			**0.43**
R13. A significant portion of mindfulness meditation is actually harmful to people’s health.	0.688			**0.59**
R14. Mindfulness meditation only works on people who believe in it.			0.578	**0.32**
R16. There is no evidence that mindfulness meditation is effective.			0.822	**0.70**
R19. Mindfulness meditation practice should be made available in all healthcare services.	−0.436			**0.49**
R20. I would recommend mindfulness meditation practice to one of my close relatives suffering from an illness.		0.384		**0.53**

*Note.* Credibility and expectancies items: C1 to C5, Representations: items R2- R20.

The first factor, which accounted for 19.3% of the total variance, comprised eight items reflecting beliefs that meditation is not adaptable to individuals’ difficulties and that it may be potentially dangerous (*α* = 0.88). The second factor explained 19.1% of the variance and included six items related to the perceived efficacy of meditation in addressing health problems (*α* = 0.89). The third factor accounted for 18% of the total variance and encompassed six items expressing beliefs that meditation is comparable to a placebo or is considered unscientific (*α* = 0.84).

### Analyses

Socio-demographic data were analysed using frequency tables to examine the repartition on study levels and occupational category. We used Chi squared tests to examine any differences between the two subsamples on health and behavioural variables and *T*-Tests to compare the two subsamples on beliefs, attitudes and motivations for the practice. The means, standard deviation and item reliability were performed with Jamovi software version 2.2.5. To address the first objective of exploring the beliefs about MM in meditators and non-meditators, we conducted two different lexicometric analyses on the free associations with IRaMuTeQ software version 0.7 alpha 2. In IRaMuTeQ, the analysis relies on word co-occurrences—that is, the tendency of certain words to appear together within the same textual segments. These co-occurrence patterns reflect underlying associations in participants’ discourse and serve as the basis for grouping segments into thematically coherent lexical classes. The first analysis, the Descending Hierarchical Classification (DHC), consists in delimiting thematic classes within the corpus according to statistically significant tests (such as Chi-squared) [53]. These classes are displayed in a tree structure (called a dendrogram), which provides insights into the content of participants’ beliefs, as well as structural relationships, semantic oppositions, or groupings among classes. All collected variables were included in the DHC analysis to determine which variables were significantly associated with each thematic class—thus addressing our second objective.

## Results

The samples were nearly comparable, and the differences in terms of socio-demographic characteristics, health and health behaviours, beliefs about MM and motivation to engage in MM between meditators and non-meditators were limited to the occupational category and to the three factors of the quantitative measure of the beliefs about MM (see Tables 2–4) for full descriptive statistics). There was significantly more self-employed participants in meditators than in non-meditators (*χ²*(10) = 11.9, *p* = .037, Cramer’s **V* *= .272). Compared to non-meditators, meditators perceived MM as less harmful and inappropriate (*t*(165) = 2.60, *p* = .01, *d* = 0.41), more effective (*t*(165) = −2.98, *p* = .003, *d* = 0.47) and associated MM with a lower placebo effect (*t*(165) = 4.50, *p* < .001, *d* = 0.72).

**Table 2:** Comparison of socio-demographic variables based on practice.

Variables: modalities	Non-meditators (n = 62) N(%)	Meditators (n = 105) N(%)	χ*²*	*p*	Cramer’s *V*
Gender: Male	11 (19.0)	18 (17.5)	1.17	.556	.085
Gender: Female	47 (81.0)	83 (80.6)	–	–	–
Gender: Non-binary	0 (0.0)	2 (1.9)	–	–	–
Studies: Certificate	0 (0.0)	2 (1.9)	2.65	.851	.128
Studies: Vocational certificate	2 (3.4)	4 (3.9)	–	–	–
Studies: Vocational aptitude certificate	0 (0.0)	1 (1.0)	–	–	–
Studies: High school diploma	16 (27.6)	27 (26.2)	–	–	–
Studies: Bachelor’s degree	21 (36.2)	34 (33.0)	–	–	–
Studies: Master’s degree	17 (27.6)	28 (27.2)	–	–	–
Studies: PhD	2 (3.4)	7 (6.8)	–	–	–
^a^OC: Self-employed	3 (5.2)	17 (16.5)	11.9	.037	.272
^a^OC: Employee	28 (48.3)	33 (32.0)	–	–	–
^a^OC: Student	24 (41.4)	35 (34.0)	–	–	–
^a^OC: Retired	0 (0.0)	5 (4.9)	–	–	–
^a^OC: Unemployed	2 (3.4)	8 (7.8)	–	–	–
^a^OC: Other	1 (1.7)	5 (4.9)	–	–	–

*Note.* Missing data: gender: n = 6; studies: n = 6; OC: n = 6. The *V* corresponds to the Cramer’s *V* effect size.

^a^OC: occupational category.

**Table 3:** Comparison of health and behavioral variables based on practice.

Variables: modalities	Non-meditators (n = 62) N(%)	Meditators (n = 105) N(%)	χ*²*	*p*	Cramer’s *V*
Tobacco use: yes	13 (22.4)	29 (28.4)	0.69	.406	.066
Tobacco use: no	45 (77.6)	73 (71.6)	–	–	–
Alcohol: never [0 times a month]	14 (22.6)	21 (20.2)	0.41	.813	.050
Alcohol: average [1–10 times a month]	29 (46.8)	54 (51.9)	–	–	–
Alcohol: regular [1–7 times a week]	19 (30.6)	29 (27.9)	–	–	–
Cannabis: never [0 times a month]	57 (91.9)	94 (90.4)	0.68	.711	.064
Cannabis: average [1–10 times a month]	4 (6.5)	6 (5.8)	–	–	–
Cannabis: regular [1–7 times a week]	1 (1.6)	4 (3.8)	–	–	–
TAC^a^: beneficial [less than 2 out of 3 regular behaviors among tobacco, alcohol and cannabis use]	54 (87.1)	90 (86.5)	0.01	.918	.008
TAC^a^:detrimental [2 or 3 out of 3 regular behaviors among tobacco, alcohol and cannabis use]	8 (12.9)	14 (13.5)	–	–	–
Vegetable and fruits: low [less than once a week, once a week or every two or three days]	21 (33.9)	23 (22.1)	2.76	.097	.129
Vegetable and fruits: high [every day or several times a day]	41 (66.1)	81 (77.9)	–	–	–
Fast food: low [less than once a week or once a week, every two or three days]	61 (98.4)	103 (99.0)	0.14	.710	.029
Fast food: high [every day or several times a day]	1 (1.6)	1 (1.0)	–	–	–
Sweets: low [less than once a week or once a week, every two or three days]	35 (56.5)	72 (69.2)	2.77	.096	.129
Sweets: high [every day or several times a day]	27 (43.5)	32 (30.8)	–	–	–
Soda: low [less than once a week or once a week, every two or three days]	54 (87.1)	87 (83.7)	0.36	.549	.047
Soda: high [every day or several times a day]	8 (12.9)	17 (16.3)	–	–	–
Nutrition: beneficial [less than 2 out of 3 high among fast food, sweets, soda consumption]	56 (90.3)	95 (91.3)	0.05	.824	.017
Nutrition: detrimental [2 or 3 out of 3 high among fast food, sweets, soda consumption]	6 (9.7)	9 (8.7)	–	–	–
Coffee and tea: low [less than three cups per day]	41 (70.7)	76 (73.8)	0.18	.672	.033
Coffee and tea: high [three cups or more per day]	17 (29.3)	27 (26.2)	–	–	–
Sleep: low [less than 7 hours per night]	14 (24.1)	19 (18.4)	–	–	–
Sleep: high [seven hours or more per night]	44 (75.9)	84 (81.6)	0.74	.390	.068
Physical activity: never	22 (33.5)	21 (20.2)	4.78	.091	.170
Physical activity: average [Once to three times a week]	33 (53.2)	67 (64.4)	–	–	–
Physical activity: regular [Four to seven times a week]	7 (11.3)	16 (15.4)	–	–	–
Chronic disease: yes	8 (13.8)	18 (17.5)	0.37	.542	.048
Chronic disease: no	50 (86.2)	85 (82.5)	–	–	–

*Note.* Missing data: tobacco use: n = 7; alcohol use: n = 1; cannabis use: n = 1; TAC n = 7; vegetable and fruits: n = 1; fastfood: n = 1; sweets: n = 1; soda: n = 1; nutrition: n = 1; coffee and tea: n = 6; sleep: n = 6; physical activity: n = 1; chronic disease: n = 6. The *V* corresponds to the Cramer’s *V* effect size.

^a^TAC: score integrating tobacco, alcohol and cannabis use.

**Table 4:** Descriptive statistics and *T*-Tests with the beliefs and motivation variables.

Variables	Non-meditators (n = 62)			Meditators (n = 105)					
	M	Mdn	*SD*	M	Mdn	*SD*	*t*	*p*	*d*
Harmful and inappropriate	0.25	0.25	1.02	−0.15	−0.5	0.96	2.60	0.01	0.41
Perceived efficacy	−0.29	−0.17	1	0.17	0.11	1	−2.98	0.003	0.47
Placebo effect	0.42	0.43	0.9	−0.25	−0.28	0.97	4.50	<.001	0.72
Self-motivation	3.73	3.73	0.60	3.90	3.91	0.56	−1.85	0.067	0.30

*Notes.* Harmful and inappropriate, Perceived efficacy and Placebo effect are the 3 factors of the Beliefs scale. Self-Motivation is the unique factor of the same scale. M: mean; Mdn: median; *SD*: standard deviation; the *t* corresponds to the *t*-value of the Independent Samples *T*-Tests. The *d* corresponds to the Cohen’s *d* effect size.

The DHC analysis processed 61.25% of the corpus’ free associations (N = 817; 5% were rated negative, 30% neutral, 65% positive) and generated 5 unlabelled lexical classes illuminating a diversity of beliefs about MM. The labelling process underwent investigators triangulation. This technique involved a first step of independent class labelling by three of the four authors, all of whom had experience in qualitative analyses. A second step entailed proposing common labels and engaging in a negotiation process to balance out the subjectivity of the interpreters and achieve a degree of convergence in the results [54].

**Fig 1:**
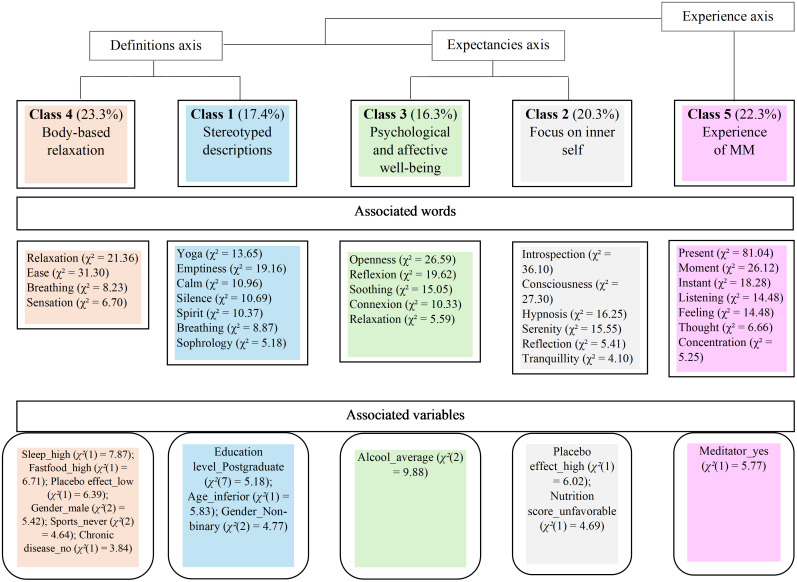
Results of the Descending Hierarchical Classification with classes and axis names and percentages for each class, most representative words and significantly associated variables (*p* < .05).

The structural organization of the beliefs was revealed through the presence of three axes (Fig 1). Fig 1 illustrates the three axes and the link between the classes and the associated variables when statistically significant (*p* < .05).

The “Definition” axis encompassed Class 1 “Stereotyped descriptions”, referring to a set of beliefs about Eastern and Western practices, atmospheres and settings, and Class 4 “Body-based relaxation”, referring to beliefs of MM as a relaxation method. Class 1 was associated with three variables: Education level_Postgraduate (*χ²*(7) = 5.18); Age_inferior (*χ²*(1) = 5.83) and Gender_Non-binary (*χ²*(2) = 4.77). Class 4 was associated with 6 variables: Sleep_high (*χ²*(1) = 7.87); Fastfood_high (*χ²*(1) = 6.71); Placebo effect_low (*χ²*(1) = 6.39); Gender_male (*χ²*(2) = 5.42); Sports_never (*χ²*(2) = 4.64) and Chronic disease_no (*χ²*(1) = 3.84).

The “Expectancies” axis encompassed Class 2 “Focus on inner-self”, alluding to a diversity of states of consciousness and an inward-looking stance, and Class 3 “Psychological and affective well-being”, alluding to desired and optimal psychological and emotional outcomes. Class 2 was associated with two variables Placebo effect_high (*χ²*(1) = 6.02) and Nutrition score_unfavorable (*χ²*(1) = 4.69). Class 3 was associated with 1 variable: Alcool_average (*χ²*(2) = 9.88).

Distinct from the four aforementioned classes, Class 5 “Experience of MM” referred to the experiential knowledge of MM. Both the “Definitions” and “Expectancies” axes were separated from the “Experience” axis, which contained only Class 5, associated with meditation practice (Meditator_yes (*χ²*(1) = 5.77). The other four classes represented knowledge and beliefs shared by both meditators and non-meditators.

## Discussion

The current study mainly aimed to explore the content of the beliefs about MM. Its novelty lies in employing a comparative approach to identify potential differences or similarities in how meditators and non-meditators view MM. Additionally, we investigated the contributions of health, belief-related, meditation and socio-demographic variables into the structure of the beliefs. Regarding the first two hypotheses, we discuss the results in terms of both what was present in the beliefs about MM (H1), what was common or different (H2), and what was absent. To answer the third hypothesis (H3), we discuss the possible meanings of each variable in association with specific classes.

Our findings were rather consistent with our first hypothesis on the predominantly positive valence of the word associated with MM, and coherent with [37] suggesting that both mindfulness and a mindful target were perceived positively. They were also in line with H1 regarding the association of MM with psychological and physical well-being (see Classes 3 and 4). However, the participants resorted to additional beliefs we labelled “stereotyped descriptions”, such as the universe of complementary therapies (e.g., sophrology, yoga) and holism (e.g., spirit) (see Class 1).

The results also brought evidence for H2. Whereas four out of five classes were consensually shared by both meditators and non-meditators, Class 5 distinctly stand out due to its content related to an experiential knowledge of MM. From a comparative standpoint, the primary distinction in beliefs between meditators (predominantly women) and non-meditators revolved around a single, almost tautological, experiential understanding. Actually, in Class 5, meditators demonstrated a significantly more accurate definitional approach to the practice, which includes present-moment awareness, concentration – a type of meditative practice (i.e., Samatha), and listening to one’s feelings (for a review on MM definitions, see [55]). Consequently, the beliefs about MM did differ based on the practice of MM, but to a lesser extent than we anticipated, as evidenced by only one class (i.e., Class 5 “Experience of MM”).

Moreover, with regards to these results, discussing what was absent from the beliefs about MM from both a critical perspective and in terms of future directions, is worthwhile. First, it appears that core elements of the most influential definitions of MM [8,56,57], such as attention, acceptance, non-judgement and non-reactivity were not evoked in either subsample. More unexpectedly, the word “health” was absent from the corpus, and there was no mention of any spiritual or religious elements, including ethical concerns, that would have been expected to emerge, given that contemporary mindfulness meditation is predominantly known to have emerged from Buddhist traditions—although some of its components are also shared by other religious or spiritual systems (for a critical review, see [27]). Most interestingly, the meditators did not produce any words related to group-based dynamics, though MM is the archetype of group-based therapy (e.g., [58,59]). Nor did they refer to the challenging or adverse aspects of the practice despite the existence of meta-analyses addressing this concern [12,13]. As for non-meditators, they did not allude to sectarianism or other stereotypes associated with the practitioners’ specific group memberships (e.g., Hippies and New Agers [60,61]).

Eventually, the exploratory hypothesis (H3) was supported by the association of multiple variables with different classes. The socio-demographic characteristics impacted the content of the beliefs (4 variables). Indeed, Class 1 “Stereotyped descriptions” was significantly associated with younger participants, those holding a PhD, and non-binary individuals. The association with younger individuals may reflect a greater reliance on social categorization and generalized representations, particularly when individuals lack direct experience with the object—such as mindfulness meditation, which tends to be practiced by older adults, as shown by evidence that meditators are on average older than non-meditators [62]. For PhD holders, this may indicate a more distanced or conceptual engagement with the topic, leading them to reproduce culturally widespread—though not necessarily negative—portrayals of mindfulness. Similarly, non-binary participants might demonstrate a form of distance toward the practice, as gender non-conforming individuals remain underrepresented in both research and clinical applications of mindfulness-based approaches [63]. Class 4 “Body-based relaxation” showed a significant relationship with men, which may reflect gender differences in how relaxation is experienced and valued. Men tend to focus more than women on physical aspects of stress relief and relaxation practices [64], possibly explaining their stronger association with body-based relaxation themes.

Health and health behaviours participated in shaping the beliefs (6 variables). A high amount of sleep, high fast-food consumption, absence of chronic disease, and never practicing sports were associated with Class 4 “Body-based relaxation.” These variables relate to bodily experiences, whether pleasant or unpleasant. Notably, eating healthier is a reason for practicing MM [1]. An average alcohol consumption was significantly associated with Class 3 “Psychological and affective well-being,” indicating that these participants may perceive mindfulness as a means to support emotional regulation and psychological balance, similar to how moderate alcohol consumption is sometimes used to cope with stress [65]. An unfavourable nutrition score had a significant relationship with Class 2 “Focus on inner self,” possibly reflecting a disengagement from physical health in favour of introspective or self-reflective aspects of mindfulness.

Ultimately, as shown in Table 4, the fact that meditators perceived MM as less harmful and inappropriate, more effective, and less likely to be a placebo than non-meditators may stem from their higher treatment credibility and expectancy, possibly because they had already undergone the intervention [66,67]. Among these Beliefs and attitudes towards MM, one of them contributed to different classes: the perception of MM as a placebo or lacking scientific credibility. Participants who did not view MM as a placebo were indicative of Class 4 “Body-based relaxation”. The perceptions of scientific efficiency for bodily matters may be influenced by the authority attributed to medical sciences over psychological sciences [68]. The association of placebo beliefs with Class 2 “Focus on inner self” class aligns with research in pain management showing that mindfulness benefits may partly stem from subjective processes like expectations, conditioning, and social cues [69]. This suggests that some participants perceive mindfulness as an internally driven, psychologically mediated phenomenon rather than a strictly scientific intervention.

Overall, these findings (H1, H2 and H3) appear to indicate three observations. First, there is a consensus perception of MM in terms of expectancies and a shared understanding of MM as an intra-individual tool for self-regulation, self-awareness and self-knowledge consistent with scientific advancements on MM mechanisms over the last decade [70–72]. A commonality in the imagery associated with the practice among both meditators and non-meditators also underlies this consensus. Second, there is a social acceptance of MM, as regards to a positive consensus towards its beneficial aspects as found in a student population [41], and facilitated by the secularization process [5]. In this consensus, the idea of a “too-much-of-a-good-thing” effect (see [73]) pending to its inevitable disavowal has not yet permeated the general population. Third, stemming from the first two observations, there is evidence of the non-integration of critical and spiritual knowledge into common-sense knowledge. This may attest to the effective transformation of MM over the last decades into a secular, universal and value-free practice as advocated by its earlier scientific proponents [74] and since its incorporation in standardized protocols to address stress-related issues [5,75]. Contrary to [26] (p. 5) who argue that “Contrary to the McMindfulness critique, common understandings of mindfulness appear to highlight engagement-related processes, rather than chiefly focusing on mindfulness-as-relief”, our findings suggest that the shared knowledge of MM within our sample was constrained to a form of regulation. However, consistent with [34] (p.11), we did identify a “certain degree of misalignment between scholarly and lay understanding of mindfulness”, primarily concerning ideological rather than methodological or definitional aspects. Indeed, the critique of MM as lacking ethics or inserting into neoliberal logics, is still limited to the academic field.

In addition to these empirical observations, we noted that the implemented variables were relevant for highlighting the organization (i.e., definitions, expectancies, experience) and social psychological logics underpinning the beliefs about MM. Indeed, despite the seemingly consensual nature of these beliefs, the results indicated subtle nuanced forms of knowledge and expectancies that varied with health and socio-economic status. The findings illustrate the way a practice such as MM serves the needs for behavioural, psychological or social adjustment on the one hand, and reciprocally, how a specific perception of MM may illuminate social membership logics on the other hand [76]. The quantitative/standardized measures, in bringing additional information to the results of the free associations which are qualitative in nature, were a suitable complementary method [77]. They were able to highlight both convergent and divergent beliefs, including placebo-related beliefs about mindfulness that participants would not have spontaneously expressed. These observations lead us to conclude by saying that the beliefs about MM are smooth and homogenous among participants at first sight, but that accurate methodological tools bring a deeper understanding of these beliefs that are socially and psychologically differentiated.

### Study limitations and future directions

A substantial limitation lies in the potential bias related to participants’ geographical location, which was not accounted for in this study. Depending on whether individuals live in urban, suburban, or rural areas, their exposure to information about mindfulness—as well as access to mindfulness-related facilities—may vary. These environmental differences can influence not only beliefs and attitudes toward mindfulness, but also general health behaviours. For instance, a study conducted in India found that adults in urban areas practice mindfulness more than their rural counterparts, a pattern attributed to differences in education levels and health status [78]. Given that mindfulness practitioners in Western contexts are often from middle- or upper-class socioeconomic backgrounds [1], it would be particularly relevant to investigate how rurality versus urbanity may shape mindfulness-related beliefs and practices in France.

In addition, the French context is characterized by significant ethnic and religious diversity [79]—dimensions that were not examined in this study, but that may introduce a cultural bias limiting the generalizability of the findings to countries with lower cultural heterogeneity or different cultural and religious frameworks. Furthermore, depending on the degree to which individuals adhere to the French ideal of *laïcité*—a culturally specific model of secularism emphasizing the strict separation of religion and state and distinct from Anglo-Saxon interpretations [80]—their perception and acceptance of mindfulness, particularly in its secularized form, may vary. Future studies would benefit from taking this ideological dimension into account, as it could prove central to understanding the way mindfulness is framed and interpreted in the broader cultural context.

With regards to the nature of the sample, two limitations should be noted. First, the subsample of non-meditators was smaller than that of meditators. However, statistical analyses revealed no significant differences between the two groups, except for occupational category. Moreover, when using a free association methodology, a smaller sample size is not particularly problematic [43]. The second limitation concerns the categorization of participants as either meditators or non-meditators. While this distinction was based on a clear criterion (i.e., whether participants had ever practiced mindfulness meditation), it does not necessarily reflect participants’ subjective identification with one group or the other. A more direct question such as “Do you identify as a meditator?” could have provided more accurate and nuanced information. Moreover, the category of “meditators” itself is heterogeneous, encompassing individuals with varying degrees of experience and commitment. To partially address this issue, we included a variable of seniority (based on the median) in our CHD analyses, allowing us to distinguish more experienced practitioners from occasional or recent ones. Importantly, the sample consisted exclusively of secular meditators, embedded in contemporary French society, with access to digital platforms and exposure to social discourses surrounding mindfulness. As such, it did not include individuals such as monastics or those practicing within isolated or traditional spiritual communities. This context likely shaped the participants’ representations of mindfulness and should be considered when interpreting the findings. Future research should more clearly investigate these identification processes at the time of inclusion.

## Conclusion

This research has fundamental implications, as it questions the current role of MM in healthcare. Our results illustrate the integration of a two-fold wave of secularization. The first wave involved the implementation of standardized health protocols, where spirituality was deliberately excluded. Now, a second wave seems to be emerging. With most beliefs distancing MM from health-related aspects, this wave appears centred on personal development, largely devoid of explicit health characteristics. This conveys the idea of an expansion of the offer beyond healthcare settings to non-ill individuals, reflecting both a completely acceptable “do-it-yourself medicalization” phenomena [81] or the takeover of self-development over health [82]. From these remarks stem practical implications, because spontaneous beliefs about MM appear to focus mainly on its positive aspects. When implementing MM in any context (including YouTube videos and Apps), ethical considerations would suggest informing participants about potential adverse effects, inefficiency, as well as the ideological and (non)-spiritual underpinnings of MM practice.

## Supporting information

S1 Table Free evocations dataset with associated variables. (TXT)

S2 Table Types of practices listed and resources used to practice. (DOCX)
