# Supplementary material for: Mindfulness beyond secularization: Beliefs across meditators and non-meditators reflect a consensus on personal development over health and spirituality
Source: PLoS One. 2025 Sep 8;20(9):e0331021. doi: 10.1371/journal.pone.0331021 (PMC12416702; doi:10.1371/journal.pone.0331021)
Supplement: S2 Table — (DOCX) [file pone.0331021.s002.docx]

**Table S2. Types of practices listed and resources used to practice.**

| **Type of practice** | **N(%)** |
| --- | --- |
| Yoga | 38 (36.2) |
| Seated meditation | 46 (43.8) |
| Standing or walking meditation | 16 (15.2) |
| Breathing meditation | 74 (70.5) |
| Other | 21 (20.0) |
| **Type of resource** |  |
| No resource | 37 (35.2) |
| App | 35 (33.3) |
| Book | 8 (7.6) |
| Video | 29 (27.6) |
| Podcast | 14 (13.3) |
| In person session | 31 (29.5) |
| Retreat | 6 (5.7) |
| Other resources | 10 (10.0) |

*Notes.* Each practice and each form of resource can be combined cumulatively with others. “Other types of practices” include meditation in different positions, prayer, mantras, sophrology, hypnosis, reflexology and energy healing sessions. “Other resources” include relaxing music, recorded audios, sessions with a sophrologist and sessions with a nurse.
